# “There is a fear that you will be attacked just for the act of working in health”: a survey of experiences of violence against healthcare in Colombia

**DOI:** 10.1186/s13031-023-00548-3

**Published:** 2023-10-24

**Authors:** Katerina Crawford☥, Tatiana Florez☥, Mario Rodriguez, Lendy Cirado, Róisín Read, Rohini Haar

**Affiliations:** 1 RIAH Consortium, University of Manchester, Stolkholm, Sweden; 2 RIAH Consortium, University of Manchester, Bogotá, Colombia; 3 University of Manchester, Humanitarian and Conflict Response Institute, Manchester, UK; 4 School of Public Health. Division of Epidemiology, University of California, Berkeley, Berkeley, CA USA

**Keywords:** Colombia, Misión Médica, Violence against healthcare, Attacks on health, Armed conflict, Healthcare, Medicine, International Humanitarian Law, Geneva conventions, War Crimes

## Abstract

**Background:**

Colombia has experienced decades of conflict between the government and non-state actors. Attacks on healthcare have been a grave but regular facet of that violence. In response, the Misión Médica (MM) program was developed to support, protect, and defend healthcare. Sporadic violence continues, with many recent attacks perpetrated not by armed actors but by residents. Given the history of conflict and ongoing violence, we sought to capture the perspectives of both healthcare workers (HCWs) and community members (CMs) regarding the characteristics and impacts of attacks on health in Colombia to gain insight into how to better prevent violence and mitigate its impacts.

**Methods:**

A cross-sectional survey was conducted from January to March 2021 in seven departments in Colombia in regions that witnessed attacks on healthcare. Questionnaires were administered to HCWs and CMs via purposive sampling, probing their experiences with attacks on health with both closed and open-ended questions. The categorical responses were stratified by health worker vs. non-health worker and descriptively analyzed. Narrative responses were analyzed via a hybrid deductive/inductive thematic approach.

**Results:**

Seventy-three individuals participated in the study (36 HCWs and 37 cm). Approximately 77% of HCWs believed that attacks on healthcare impacted health outcomes while 68% of CMs did not see a direct connection between violence against healthcare and poor health outcomes. Awareness of the MM program was significantly different between HCWs (83.3%) and CMs (37.8%). The survey responses explored the characteristics of attacks on health, compounded impacts of violence on the health system, personal impacts, and perspectives on mitigation efforts.

**Conclusions:**

The study demonstrates that: (1) attacks on healthcare are context-dependent and require a local lens for mitigation and management; (2) both HCWs and CMs have critical perspectives that must be considered, (3) the impacts of violence against healthcare are complex and compounded and (4) that awareness of the legal protections of the Geneva Conventions must be combined with education on the health impacts for robust protection strategies. Critically, Both CMs and HCWs experience fear and psychosocial ramifications of these attacks, suggesting the need for stronger protections and resources to support the health workforce and the local community.

## Background

Despite the clear protections afforded to healthcare under International Humanitarian Law (IHL) [1], attacks on healthcare are a grave but common occurrence during armed conflicts across the world. In Colombia, the site of the longest lasting conflict in the Western Hemisphere [2], these attacks, including interference with healthcare services, kidnappings, intimidation and violence against health workers, and seizure of medical supplies and facilities, have been reported throughout the more than 50-year conflict [2].

### Attacks on healthcare and their impacts

Despite growing concern around attacks on healthcare worldwide [3–6], data from Colombia and other conflict-affected countries has been limited. However, the growing numbers of incidents recorded by the Safeguarding Health in Conflict Coalition and Insecurity Insight underscore that the problem is much more frequent and profound than previous reporting would suggest [7]. 2022 data from Insecurity Insight has reported nearly 2000 incidents across the globe and 2023 is likely to have even more recorded [8]. While evidence on the methods and need for documentation is developing, very little is still understood about the short, medium and long term impacts of the violence on health. Both in Colombia and globally, focus on legal violations and immediate consequences has overshadowed assessment of the broader and longer-term impacts on health workers and the health system, and even fewer combine community member perspectives with those of HCWs. However, a deeper understanding of these impacts could reinforce advocacy efforts for preventing attacks and holding perpetrators accountable, as well as targeting recovery work to those most impacted. The Researching Impacts of Attacks on Health consortium aims to explore the impacts of violence against healthcare in conflict in conflicts in Afghanistan, Myanmar, Nepal, and Central African Republic as well as in Colombia [9].

### Conflict in Colombia and the Misión Médica

The modern roots of Colombia’s long conflict have resulted in complex and violent dynamics that linger today. In the 1960s, guerrilla groups started to take hold in Colombia, beginning with the rise of the Revolutionary Armed Forces of Colombia (Las Fuerzas Armadas Revolucionarias de Colombia) (FARC) in 1964, and the nascent arm of the National Liberation Army (Ejército de Liberación Nacional) (ELN) in 1965, among other left-wing movements [10]. The introduction of the drug trafficking trade in the 1970s exacerbated the violence between government forces and non-state actors and shifted the balance of power towards the drug cartels who, in the 1980s, helped found right-wing paramilitary groups to protect their interests [10]. As paramilitary groups consolidated power under the Self-Defense Forces of Colombia (Autodefensas Unidas de Colombia) (AUC) [11], the Colombian government found itself in conflict with both guerilla groups and paramilitaries for decades. The 2003–2006 demobilization of the AUC and the 2016 peace accord between the Colombian government and the Revolutionary Armed Forces of Colombia-People’s Army (Las Fuerzas Armadas Revolucionarias de Colombia Ejército del Pueblo) (FARC-EP) were followed by a gradual reduction in the intensity of the conflict [11, 12]. During the most turbulent phase of the modern conflict, groups involved committed numerous violations of IHL, particularly against civilians, including forced disappearances, massacres, mass forced displacement, terrorism, kidnappings, torture, extrajudicial executions, and attacks against health workers, among other war crimes [13–17].

The Colombian government began monitoring attacks against healthcare infrastructure and personnel in 1998 [18]. As of 2019, 2,419 attacks have been documented, according to data shared by the Colombian Ministry of Health staff (as of January 2020). While these attacks occurred across the nation, according to discussions with local healthcare workers (HCWs), healthcare infrastructure was particularly affected in regions that were hotspots for the armed conflict– including the Valle del Cauca, Arauca, Norte de Santander, Antioquia, Meta, and Nariño departments.

To address the prevalence of attacks against Colombia’s healthcare infrastructure—as well as the adverse effects on the delivery of health services—the Ministry of Health and Social Protection developed the Misión Médica (“Medical Mission’’) program via Resolution 1020 of 2002, and in 2012 established the manual of the Misión Médica (MM) and the regulations for use of the emblem through Resolution 4481 [19]. The purpose of the Misión Médica emblem is to identify persons, places, organizations, and materials associated with the rendering of medical services, with the goal of increasing awareness of their protected status and reducing the incidence and severity of attacks on the healthcare system [19]. In effect, the Ministry of Health labels all healthcare personnel and services in Colombia with Misión Médica’s emblem, alongside an ongoing awareness campaign for both local communities and health workers designed to make explicit the protections guaranteed to HCWs and infrastructure by Colombian and international laws [19]. Furthermore, Misión Médica collaborates with other organizations in Colombia to ensure that any individuals who are providing health services to the population are provided with the emblem and are thus recognized as a protected group. Specifically, Misión Médica supports the Regulatory Center for Emergencies (Centros Reguladores de Emergencias y Desastres) (CRUE) which is the entity responsible for all management and coordination of population health following an emergency or disaster [20].

Despite these efforts, violence against healthcare in Colombia persists and is attributed to a variety of perpetrators, including local residents and non-state armed groups [21]. The COVID-19 pandemic deteriorated the relationship between community members (CMs) and HCWs, a phenomenon reported in many countries worldwide [22, 23]. In 2020, Colombia experienced the greatest number of attacks on healthcare in the past 24 years due to the compounded stress of the COVID-19 pandemic [21], demonstrating the need for further investigation on how to mitigate the violence.

### The knowledge gap

Researching the impacts of the complex and chronic violence against healthcare in Colombia takes us closer to a better understanding of violence against healthcare globally and nationally. Particularly important is how HCWs and community members have been impacted by attacks on healthcare and the effects on the health system. The responses of HCWs to the violence in Colombia play a key role in mitigating the severity of these impacts and they could share lessons with other medical professionals and humanitarian workers facing similar threats across the globe. Perspectives from community members are rarely elicited regarding these attacks but could be a critical component to a deeper understanding of the drivers of violence, particularly when growing numbers of attacks are reportedly perpetrated by local residents. This study aims to contribute a more nuanced and wider lens into attacks on health in Colombia and their impacts on HCWs and the community as well as perceptions of both HCWs and CMs regarding the Misión Médica campaign. The evidence base can also inform reconstruction efforts in Colombia, specifically by identifying points of weakness in health services and focusing local and international attention on vulnerable communities.

## Methods

### Research design

This cross-sectional survey-based study conducted in seven Colombian departments aimed to understand the impacts of attacks against healthcare on health workers, community members (CMs), and the healthcare system as a whole. We utilized a semi-structured questionnaire probing the experiences of HCWs and CMs in Colombia– specifically, investigating the effect that the attacks have had on their lives, and their perceptions of the Misión Médica campaign initiated by the Colombian Ministry of Health and Social Protection. Thus, this study includes the following research questions:

1. What are the experiences of health workers and community members with attacks on health and how have these groups perceived that healthcare in Colombia has been impacted by this violence?
2. What are the perceptions of health workers and community members of the Misión Médica campaign?

**Fig. 1:**
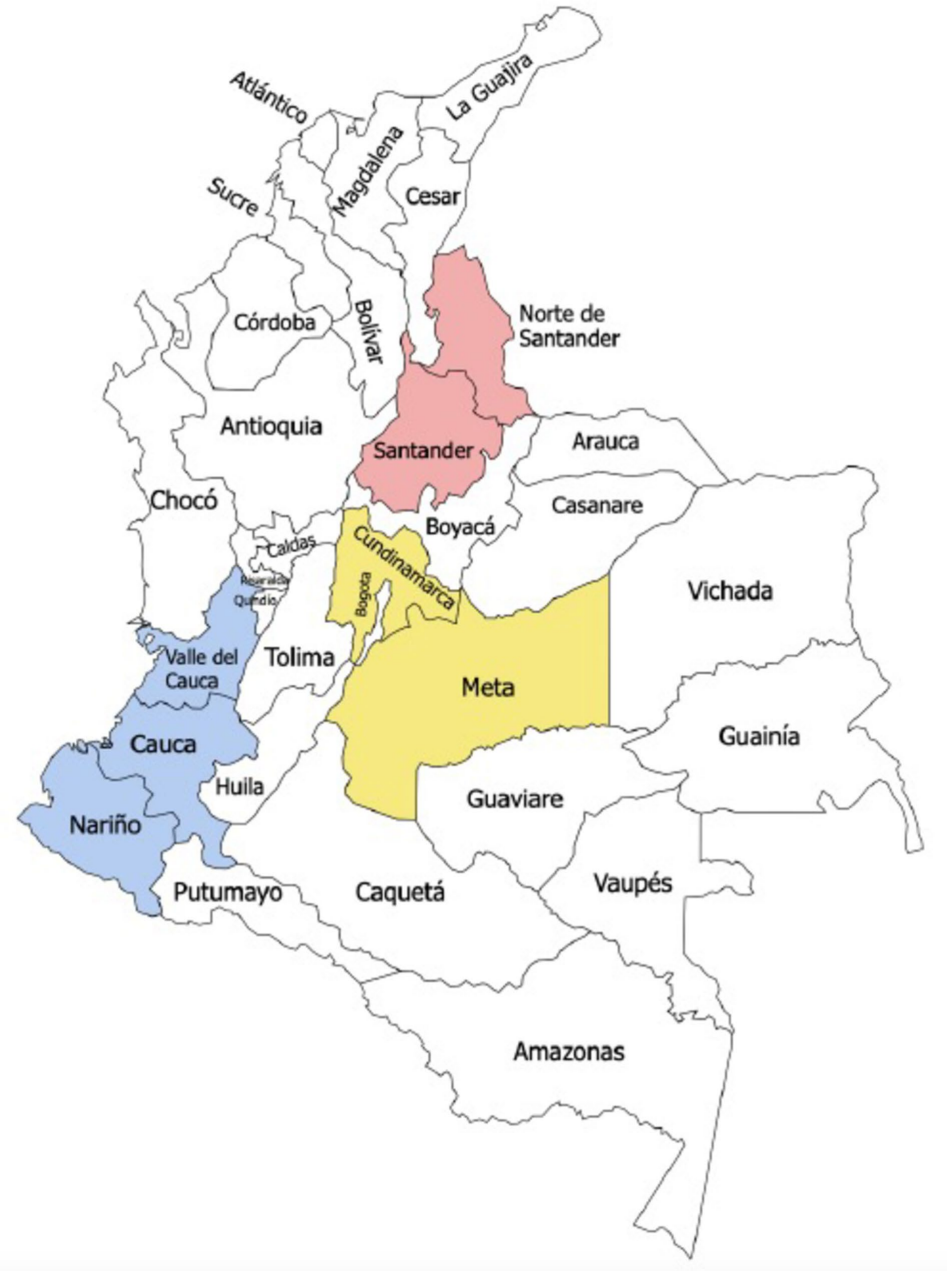
Map of Colombia with the seven study departments colored by geographic zone

The departments included in the study setting were selected due to their location within the three geographic zones with the highest frequency of attacks against health infrastructure as reported by the Ministry of Health and Social Protection (personal communication with Ministry of Health staff, January 2022) (Fig. 1):

- Zone 1 (Southwest): **Cauca**, **Nariño**, **Valle del Cauca** departments;
- Zone 2 (Northeast): **Arauca, Norte de Santander**, and **Santander** departments;
- Zone 3 (Central): **Antioquia**, **Cundinamarca**, and **Meta** departments, and the district of **Bogotá**.

### Participant selection

Inclusion criteria selected HCWs who were over the age of 18, citizens of Colombia, spoke Spanish, had worked in Colombia for at least two years, and, at the time of the study, were employed at a healthcare facility (HCF) in one of the seven departments. To get a more holistic understanding of the situation concerning the dynamic between the health sector and residents, CMs fitting the previously stated age, citizenship, and language profile, residing in the areas of interest, were also asked to take part in the study. Given the dominance of male perspectives included in conflict research on this topic [24], we recruited women and wanted at least half of the participants to be female. We also recruited a diverse set of participants from various professional and social backgrounds in the aims of learning of the myriad facets of the impacts of attacks that they may face and in aggregate, gaining insight into a broader framework for understanding these impacts. We included participants who did not directly witness or experience violence as they still lived and worked in settings with the risk of violence and we wanted to understand how the risk impacted them.

The COVID-19 pandemic coupled with an unstable security situation constrained sampling due to the need to maintain the health and physical safety of the research team and participants. For these reasons, we purposely chose sites of interviews and used convenience sampling to select HCWs and CMs who were willing and able to be interviewed in the seven departments in compliance with COVID-19 safety protocols in place at that time, including masking and social distancing.

To recruit HCWs, we coordinated with local public health authorities, prior to the study team’s travel to each department, to get permission for participant recruitment at HCFs. Potential participants were approached in the local district hospitals and clinics advised by the department’s public health authorities to contact the interviewers. Snowball sampling was used to interview additional HCWs who may not have heard about the survey, or who worked in private or more inaccessible sites.

A sample of community members were recruited at HCF sites where HCW surveys were already being conducted via the same process. Investigators approached local shop owners, families waiting in the clinic areas, and non-HCW staff for recruitment generally and interested individuals were asked to contact the interviewers.

In addition to conducting a security assessment of every study site, in all the seven departments, investigators contacted local and municipal leadership as well as hospital administration for consent to be included in the study. If affirmed, on a day specified by the local administrator, investigators traveled to a site, and met with interested staff to share the purposes of the study. Health workers at each location as well as community members who met the inclusion criteria were invited to participate.

### Data collection and analysis

Once a participant gave informed consent, research team members verbally conducted the survey, available on Github, [25] and manually recorded answers on a paper form. The semi-structured questionnaire, developed in collaboration with local partners, contained basic demographic questions, followed by questions on five themes concerning both HCWs and CMs: (1) experience with conflict and attacks on healthcare, (2) personal impact secondary to attacks, (3) professional impact secondary to attacks, (4) view or insights into impacts on the health system and (5) reflections on mitigation efforts and strategies. We used the same interview guide for both health workers and community members but adapted probes and follow up questions to be personalized to the identities, experiences and responses of the participants. Responses to open-ended questions were enriched with probes and follow up questions to gain deeper insights and describe experiences. While we did not record interviews for security and trust concerns, interviewers were trained to record as much of the responses as possible verbatim.

Sampling for a location was complete when the time allowed to conduct the survey (1–3 days) had elapsed or when the researchers felt that saturation of responses had been achieved. All interviews for the study were done from January 15 to March 30, 2021.

Survey papers were securely stored and transcribed using a secure, encrypted online software. We transferred participants’ data from paper forms into detailed spreadsheets and then translated the responses to English to facilitate analysis.

#### Quantitative analysis

Categorical data on attacks, experiences, and participant characteristics from the master spreadsheet were cleaned and reformatted prior to being uploaded to Stata [26]. First, the description of the respondents was generated for the following independent variables: sex (male, female), age (25–30, 31–40, 41–50, 51–60), job category (HCW, non-HCW), occupation, location, “been a victim/witness to an attack against MM” (yes, no). Sex, job category, and “been a victim/witness to an attack against MM” were binary variables, age was grouped into four categories, occupation was a list of 26 professions, and location referred to the seven different departments studied. Any missing data was excluded from analysis and the percentages developed reflected the relevant response divided by the total number of data points available.

Categorical survey responses were stratified by job category (HCW, non-HCW) to examine the differences in the perceptions and experiences of these two groups. All the close-ended survey questions were binary (yes, no) except for “How effective is Misión Médica?” with a categorical scale of six options (not effective, neutral, slightly effective, effective, very effective, does not know).

#### Qualitative analysis

For the narrative responses, a hybrid deductive/inductive approach [27, 28] was applied. This process was done iteratively, first with deductive codes grounded in the initial survey organization, and then inductively based on the responses within the existing codes. The *a priori* deductive codes categorized the responses into four domains: (A) characteristics of attacks on healthcare, (B) impacts of violence on the health system, (C) personal impacts, and (D) perspectives on mitigation efforts. Inductive codes were developed during the analysis process by reading and extracting salient points within the responses and categorizing them thematically. To minimize subjectivity, two researchers (KC, RH) coded narrative responses independently and came together to develop categories that fit within the previously established domains and discussed emerging themes. All inconsistencies between the codes created by the researchers were resolved by consensus. Qualitative analysis was conducted on the Dedoose application [29].

### Ethical considerations

Ethical approval for this study was granted by the Human Research Protection Program at the University of California, Berkeley (Protocol ID# 2020-12-13895).

Our global research team worked closely with our national researchers when developing the study and creating the survey to assure it was context appropriate and to reduce any negative risk to the study participants and local researchers or to re-entrench historical power dynamics. The partnership among researchers was essential to ensuring that the study was developed with the aim to catalyze positive change for both HCWs and CMs. Moreover, for the members of our research team who were not from the cities studied, particularly those who were involved with data analysis but living outside of Colombia, discussions on and practice of reflexivity [30] aided the team in attempting to reduce bias.

## Results

### Participant characteristics

Of the 73 people interviewed, 42.5% were male and 57.5% were female. Most respondents were between the ages of 31 and 50 (71.6%). Almost half of the interviewees were employed in the healthcare field (49.3%) and of those 36 HCWs, most worked as nurses (47.2%), doctors (19.4%) and operating room technicians (5.6%). There were 37 cm sampled who held a variety of occupations including non-healthcare administrators (13.5%), drivers (8.1%), mechanics (8.1%) and engineers (8.1%). The respondents worked in seven different departments across Colombia with the majority residing in Norte de Santander (24.6%) and Cauca (16.4%). 30 people (41.7%) said they had been a victim or witness to an attack against healthcare. Demographic information about the interviewees can be found in Table 1.

**Table 1:** Description of the interviewees

Independent variable Category	N=73
	n (%)
**Sex**	
Male	31 (42.5)
Female	42 (57.5)
**Age (years)**	
25–30	12 (17.9)
31–40	25 (37.3)
41–50	23 (34.3)
51–60	7 (10.5)
**Job Category**	
Healthcare	36 (49.3)
Non-healthcare	37 (50.7)
**Location**	
Cauca	12 (16.4)
Cundinamarca	3 (4.1)
Meta	7 (9.6)
Nariño	11 (15.1)
Norte de Santander	18 (24.6)
Santander	11 (15.1)
Valle del Cauca	11 (15.1)
**Been a victim/witness to attack against MM**	
Yes	30 (41.7)
No	42 (58.3)

### Descriptive and comparative analysis

Regarding the experiences of attacks on health, 21 HCWs interviewed had either witnessed or been a victim of an attack on healthcare (60.0%) compared to nine of the CMs (24.3%). Although only 37.1% of HCWs thought that attacks impacted healthcare facilities, 77.1% of HCWs believed that attacks impacted health outcomes for the patients they serve. In contrast, most CMs did not think that attacks on healthcare impacted health outcomes (67.7%). There was also a difference between HCWs and CMs on perceptions of attacks impacting personal life. While 58.8% of HCWs believed that attacks impacted their personal lives, only 12.1% of CMs shared the same opinion.

Concerning overall awareness of mitigation efforts, 83.3% of HCWs said that they knew about the Misión Médica, compared to 37.8% of CMs. The majority of HCWs interviewed had participated in MM training (76.5%). Finally, most of the HCWs stated that MM was effective (45.2%) or very effective (19.4%) on a Likert scale. A comparison of the responses from HCWs and CMs is displayed in Table 2. Given the small sample size, significance testing was not conducted between the groups.

**Table 2:** Interview responses, stratified by HCW vs. non-HCW

*Question*	HCW	CM
Response	**N = 36**	**N = 37**
	n (%)	n (%)
***Have you witnessed or been the victim of an attack on healthcare?***		
Yes	21 (60.0)	9 (24.3)
No	14 (40.0)	28 (75.7)
***Have attacks impacted health outcomes?***		
Yes	27 (77.1)	10 (32.3)
No	8 (22.9)	21 (67.7)
***Have attacks impacted any HCFs?***		
Yes	13 (37.1)	1 (3.3)
No	22 (62.9)	29 (96.7)
***Have attacks impacted healthcare related work?***		
Yes	20 (58.8)	3 (11.5)
No	14 (41.2)	23 (88.5)
***Have attacks impacted your personal life?***		
Yes	12 (35.3)	4 (12.1)
No	22 (64.7)	29 (87.9)
***Do you know about Misión Médica?***		
Yes	30 (83.3)	14 (37.8)
No	6 (16.7)	23 (62.2)
***Do you think the current national regulations on the issue of Misión Médica are applied?***		
Yes	20 (66.7)	4 (36.4)
No	10 (33.3)	7 (63.6)
***Have you participated in Misión Médica training?***		
Yes	26 (76.5)	7 (38.9)
No	8 (23.5)	11 (61.1)
***How Effective is the Misión Médica?***		
Not effective	1 (3.2)	1 (10.0)
Neutral	6 (19.4)	3 (30.0)
Slightly Effective	4 (12.9)	0 (0.0)
Effective	14 (45.2)	4 (40.0)
Very effective	6 (19.4)	1 (10.0)
Does not know	0 (0.0)	1 (10.0)

### Qualitative analysis

The survey also asked several open-ended questions where interviewees had an opportunity to describe their experiences. Deductive and inductive analysis of the responses identified four major themes: (A) characteristics of attacks on health, (B) compounded impacts of violence on the health system, (C) personal impacts, and (D) perspectives on mitigation efforts.

#### Characteristics of attacks on health

Participants’ description of characteristics of attacks on health included perspectives on (1) perpetrators, (2) types of attacks, and (3) targets of attacks.

##### Perpetrators

Based on the interview responses, there were two main types of perpetrators identified: armed groups and community members.

Due to the sensitive nature of the subject and security concerns, we did not ask the interviewees to identify the armed groups, their motives or the methods used and thus no information on specific actors was recorded. According to the respondents, the armed groups terrorized health workers and attacked health facilities to instill fear and control the population in the relevant area. Although the armed groups rarely specifically targeted patients, they made it difficult for the community to seek healthcare or for HCWs to access patients. This sentiment was echoed by one unemployed resident from Nariño describing roadblocks en route to medical facilities: *“Always at night between 7PM and 6AM, you cannot go out because the [armed] group plants landmines in the highway.”*

Contrastingly, some respondents reported that family members of patients would become verbally or physically abusive if a HCW did not allow them to visit a patient, especially during the early months of the pandemic due to social distancing measures. In one case, an individual accompanying a patient seized a HCW as a form of leverage to ensure that the patient received care immediately. Patients and their families, at times, were violent after receiving what they perceived to be low quality healthcare from the staff at a HCF, such as the case reported by a nursing assistant from Villavicencio: *“I was leaving respiratory triage and saw a woman stretched out on the ground and I told her she should get up as the ground was dirty, and that we would bring her a seat from the waiting room. I left the patient in the chair so I could go and bring a stretcher, when I came back with the stretcher the woman got up from the chair and fell to the ground. Her two brothers then came and assaulted me.”*

*Many participants noted that these two types of violence are interconnected and mutually perpetuating. Persistent armed conflict fostered an environment in which violence is normalized and this may have emboldened community members to resort to violence, particularly against health workers who are the frequent targets or armed groups.*

##### Types of attacks

Frequently reported methods for attacks on health were physical assault, verbal assault, detainment/hostage taking, vandalism/destruction of healthcare property, and use of weapons.

Physical assault was most prevalent and was largely skewed towards HCWs with a few cases of patients being harmed. One main reason discussed during the interviews for HCWs being physically attacked was as retribution for not saving the life of a patient. However, some of the violence carried out by civilians was specifically motivated by community distrust of HCWs and a lack of education concerning the COVID-19 pandemic and the transmission of the SARS-CoV-2 virus. HCWs were often perceived as “infectious” themselves or purposefully working to spread the virus which led to them being physically or verbally assaulted. For example, a nurse from Norte de Santander detailed her experience as part of a traveling vaccination clinic: *“While leaving to vaccinate neighborhoods… a group came out, insulted us, and said that we were carrying the [COVID-19] virus in a thermos and did not permit us to vaccinate.”*

Verbal assault as well as threats, mostly by armed groups, were also frequently mentioned by interviewees. These often occurred along with detainment of HCWs and seizure of medical equipment. One nurse from Cauca described her experience: *“[There were] threats and detention for more than 3 hours of healthcare workers, patients, health center, and transport.”*

Detention and hostage-taking of HCWs by armed groups was sometimes coupled with ransom demands. A public health coordinator from Norte de Santander discussed such an attack: *“When [the ambulance] arrived at the scene, the group stopped the ambulance and demanded money. The medical personnel were held hostage for one day, and calls were made to the hospital management and families attempting to obtain a ransom for them.”*

The weapons most often described by the interviewees were guns and bombs and were generally employed by armed groups. Bombings occurred in HCFs while shootings more often happened at traveling ambulances. A community member interviewed in Cauca gave this account of an event in her hometown: *“In this area there are shootings; one day they threw a bomb at the health post of [Cauca] and there were injur[ies] among the healthcare workers.”*

##### Targets of attacks

Although HCWs were the most common targets for violence, patients, ambulances as well as HCFs were also attacked.

Given the accounts of interviewees, attacks on patients appeared to be targeted, and in the several described cases, the patient was ultimately killed. A health professional in Meta described such an instance: *“One time an ambulance was stopped and the injured person who was inside was taken. Eight days later this person was found dead.”*

Ambulances were also noted as frequent targets for attacks by armed groups. The vehicles were often stopped and the medical personnel inside were either taken hostage or robbed. There was one case of an ambulance being seized, possibly for the equipment inside, while two other interviewees reported ambulances being shot at, resulting in casualties.

Finally, vandalism was the most frequently named offense against HCFs with some members of armed groups destroying doors, chairs, and threatening HCWs with sharp objects.

#### Compounded impacts of violence on the health system

The impact of violence on the health system fell under two sub-themes including (1) workforce impact and (2) infrastructure impact.

##### Workforce impact

HCWs were most frequently impacted by the attacks among our interviewees. The detrimental effects against health personnel were concentrated in several issues: disruptions to traveling clinics/professional activities, HCW shortages, HCW job dissatisfaction, and death/injury of HCWs.

Respondents said armed groups targeted traveling clinics and HCWs due to their isolation and vulnerability. The decreased safety for HCWs outside of HCFs caused some traveling clinics to be very limited in their reach. This resulted in many areas, particularly rural towns, being without healthcare for periods of time. One nurse from Nariño described this situation: *“In the past, events that had occurred have left various towns without healthcare workers. In [my rural area], for one month, traveling services were suspended.”*

Furthermore, the absence of protection for HCWs has led to significant job dissatisfaction and, consequently, staff attrition. Several HCWs described their concern for their lives while working in the health sector. A doctor from Nariño described the poor support for HCWs as an institutional issue: *“There is a great abandonment by the state of the health sector, we do not feel safe.”*

Moreover, the injury and deaths of several health personnel have caused other HCWs to reconsider their choice of profession and some medical staff are reluctant to return to work following an attack. Community members also seem to gain an awareness of the dangers faced by HCWs from reports in local media. As stated by an administrator from Girardot: *“What I’ve heard in the news [is that] that they shot at an ambulance and killed a nurse.”*

##### Infrastructure impact

Attacks have also caused major harm to medical infrastructure through the disruption of the operation of HCFs as well as interference in the delivery of medication and supplies.

The activities of armed groups have caused HCFs to shut down entirely for periods of time, as described by a driver from Santander: *“In 2010 when I lived in [rural area], one time, armed men came, informing us that they wanted to close the health center because a 2-day curfew had been initiated the zone.”*

Moreover, stealing of medical supplies and equipment caused serious delays, or in some cases, complete lack of access to healthcare. In Meta, a health professional stated: *“They [armed group] left us without an ambulance for 2 month[s].”* A community member from Cundinamarca described an incident of vaccine theft: *“Vaccines were stolen from the rural area. I cannot be vaccinated. I think the groups are aware that they should vaccinate. They steal the vaccine.”*

#### Personal impacts

The interviewees described the effects on their personal lives as falling into either (1) mental distress or (2) behavioral adaptations.

##### Mental distress

Both HCWs and community members who experienced attacks reported signs of mental distress such as fear and insomnia. Many respondents described a constant fear that interfered with their personal lives and overall quality of life. One nurse from Meta stated: *“Many times when you go out on the street, there is a fear that you will be attacked just for the act of working in health”*.

A nursing assistant from Meta described the mental toll of an attack: *“The attack had a psychological impact. I was disabled for 7 days.”*

##### Behavioral adaptations

The lack of safety, exacerbated by the negative mental effects of the attacks, prompted behavioral changes among many HCWs and community members, including adaptation/normalization to the experience of conflict, relocation, and reluctance to discuss attacks.

Several interviewees described the attacks on healthcare as a normal part of life which have been occurring for many years. One driver from Santander stated *“…People who live in zones of armed conflict adapt themselves and become accustomed to live in the context of conflict.”*

One HCW explained that, for mental health reasons, he decided to move away from his original place of residence following an attack. Two interviewees who had lived through attacks said that they would rather not discuss the details of what happened and that it was, overall, better not to talk about the violence against healthcare they had experienced.

#### Perspectives on mitigation efforts

Perspectives on mitigation efforts fell into two sub-themes: (1) community awareness and engagement, and (2) ongoing challenges.

##### Community awareness and engagement

Interviewees noted that protecting healthcare against attacks is challenging–there is sometimes tension between the community and health workers and a lack of respect for healthcare. Concerns about misinformation on COVID-19 and false reporting about HCWs misdiagnosing patients for financial gain was cited several times as a cause for deep concern. A nurse from Villavicencio highlighted this concern by noting, *“There are many comments on social networks that health entities want… patients, diagnosing them all as COVID.”*

On mitigation measures, several HCWs surveyed pointed to the Misión Médica (MM) campaign as the most important awareness raising and protection effort available. The MM was described as increasing access to healthcare and collaborating well with the Regulatory Center for Emergencies (Centros Reguladores de Urgencias y Emergencias y Desastres) (CRUE)-- the agency responsible for healthcare during crisis management. Other respondents described the efficacy of the MM as the organization’s ability to manage difficult situations, particularly through its partnership with the CRUE. This was perceived as particularly important during the COVID-19 pandemic. A nurse from Cauca described this collaboration: *“[The Misión Médica] has a great positive impact, effective transfer of patients in coordination with CRUE.”*

However, many HCWs and some CMs surveyed also stated that the main challenge with improving the situation of frequent attacks on healthcare was a very low level of awareness among the community concerning what the MM is and thus the need to protect HCWs. Several interviewees stated that there needs to be improved communication and education on the community level about the MM, suggesting dissemination modes such as television ads and educational pamphlets distributed in hospitals. One student from Norte de Santander stated: *“It is important to continue strengthening the topic in the community and in larger cities as the general public is not aware of it.”*

##### Ongoing challenges

In order to strengthen the MM and improve the relationship between HCWs and the community, the interviewees stated that the following critical improvements need to be made: increase protection for HCWs, raise community engagement, provide more training, expand education, and develop collaborations with other organizations.

Given the persistent targeting of HCWs in attacks, many interviewees stated that HCWs need to be more protected legally as well as physically and psychosocially. One individual suggested increasing psycho-social support and another thought having security personnel accompany HCWs in the field could be helpful. A nurse from Norte de Santander provided her perspective:

It [the Misión Médica] must be improved a lot, including a revision of the relevant legal protections, given that the latter has not added to the respect [for healthcare needed by HCWs].

Bolstering the relationship between the community and the MM was also perceived as a priority. A doctor from Norte de Santander described that there, *“must be a continuation of strength with a focus on humanization of the community.”*

Along with increasing training and education both for community members and HCWs, several respondents said that it would be beneficial for the MM to create partnerships with organizations outside of CRUE. A doctor from Santander spoke about this issue: *“The MM must improve but [be] more oriented towards other institutions related to health, such as transit and surveillance.”*

## Discussion

This study contributes to understanding the experiences and perspectives of local health workers and community members regarding violence against healthcare in Colombia. To our knowledge, this is the first study in which both healthcare personnel and community members (CMs) were interviewed in Colombia or elsewhere to understand the causes, characteristics, and impacts of attacks on healthcare. While their voices are often missing in the debates on this issue, their engagement is critical to addressing the impacts of violence as well as prevention of and accountability for the violence. This paper aimed to describe their experiences and unique perspectives with survey data as well as qualitative responses that underscored the complex personal and professional challenges that health workers and community members face in Colombia secondary to this targeted violence.

Synthesis of the responses from healthcare workers and community members yielded four key conclusions: (1) conflict-related and community violence are interlinked; (2) both health workers and community voices must be centered, and (3) the impacts of violence against healthcare are complex and compounded and (4) insights must be operationalized.

### Conflict-related and community violence are interdependent

According to the study results, the perpetrators of healthcare violence in Colombia are both non-state armed groups and community members. While community member attacks are isolated incidents, generally related to the care of a loved one, non-state armed groups act deliberately to block access to HCFs and medical supplies, steal healthcare equipment, and terrorize HCWs to assert control over a certain region. The various methods used to intimidate and harm HCWs, reported on in this study, include physical and verbal assault, hostage taking, vandalism/destruction of property, and use of weapons. HCWs that are employed in traveling clinics are often attacked in remote areas. Ambulances and other healthcare vehicles are frequently targets of shootings and theft by armed groups. Less frequently, HCFs were bombed, resulting in injuries of health personnel. These methods of violence are similar to those witnessed in globally particularly in terms of the actions of armed groups [8, 24, 31]. One notable difference is that in Colombia, it has been reported that community members play a significant role in perpetrating attacks. In 2021, the ICRC reported that, in Colombia, 66% of attacks on healthcare were conducted by CMs, patients and their families while 20% were carried out by non-state armed actors [32].

The conflict in Colombia is distinguished by its timeline and intensity: it has spanned five decades and has had long and lasting impacts on all facets of the community. The attacks on healthcare by both CMs and by armed groups entrench the acceptability of violence and compound the impacts on health. The health dimension of the conflict is thus deeply embedded in the conflict. The decades of fighting have also, in some ways, normalized local violence and undermined the protected status of HCWs, as seen by the growing number of incidents of community member attacks on healthcare during the COVID-19 pandemic. The greatest number of attacks on healthcare in Colombia were reported in 2021 and were a 70% increase from the number of reported incidents the year prior [32]. The COVID-19 pandemic likely contributed to this rise in attacks on healthcare by worsening tensions between HCWs and CMs.

According to interviewee statements, pervasive disinformation as well as fear of the virus framed HCWs not as a source of help for CMs but rather as modes of viral transmission. Several verbal and physical assaults of HCWs, described by interviewees in this study, were motivated specifically by a distrust of health personnel and the misconception that HCWs were deliberately passing COVID-19 to the community. Similar patterns of community violence against HCWs appeared in India and Mexico during the COVID-19 pandemic [22]. When creating policy, it is crucial to distinguish conflict vs. non-conflict violence; however, in day-to-day work, it is exceedingly difficult to make these distinctions, particularly when analyzing the first-hand experiences of residents in conflict settings.

### Community and health worker voices must be centered

This paper is unique in engaging both local health workers and community members (CMs) to better understand the characteristics of attacks on health and their impacts in Colombia. Particularly as CMs are both perpetrators and victims of attacks on healthcare, it was critical that their perspectives were highlighted to increase understanding of the societal dynamics within Colombia.

In this study, 60% of HCWs interviewed had witnessed or been a victim of an attack on health compared to 24% of CMs, demonstrating that although both groups are affected by the attacks, healthcare staff among our cohort experienced high levels of violence. Furthermore, the majority of HCWs (77%) thought that attacks impacted health outcomes while 68% of CMs believed the opposite to be true. This may indicate that CMs are unaware of the deep impacts that attacks on health have on HCWs and facilities. Also, 83% of HCWs stated that they knew about the Misión Médica compared to 38% of CMs. This statistic reveals a key issue in the relationship between community members and health workers– there exists a lack of awareness and education on behalf of community members concerning the importance of the medical mission and the need to respect healthcare personnel. HCWs themselves and community members described a need for the community to be more involved and understanding of the challenges that HCWs face.

Underreporting of attacks on healthcare is also an ongoing problem in several countries, including Colombia and South Sudan [32, 33], partly due to normalization as well as other factors, such as lack of access and poor data collection. In South Sudan, lower-level incidents were likely not reported because they were attributed to patients trying to access treatment rather than HCWs providing care [33]. In Colombia, based on the responses of the interviewees, fear of future retribution by armed groups is likely a motivation for victims to stay silent.

The ideas laid out by the “ICRC Institutional Health Care in Danger Strategy 2020–2022” may serve as an excellent base for the community building work that is needed in Colombia. Specifically, in response to violence from the civilian population, “the ICRC will carry out behavioral-change campaigns addressing the attitudes and behavioral patterns underlying such violence” [34]. Emphasis should be placed on programs which not only educate community members about the MM but also encourage actionable shifts in the relationship between CMs and HCWs.

### The impacts are complex and compounded

The most reported personal impact of the violence against healthcare in Colombia is a persistent fear both on the part of HCWs and CMs. HCWs interviewed described living in fear due to their work in the medical sector while some community members stated they were afraid to leave their homes to access healthcare. This is a sentiment echoed by residents in Syria who have experienced attacks on healthcare for the past 11 years [35]. In the latest IRC reports, 50% of interviewees in Aleppo and Idlib (2020) were scared to seek medical care [35]. In Nigeria, health workers interviewed described similar feelings, with 80% of respondents stating that attacks on health had negatively impacted their mental wellbeing and increased their anxiety [36]. Furthermore, safety for HCWs was also a large concern–1 in 6 said they had concerns for their safety when operating in their professional capacities [36].

This fear manifests in certain behavioral adaptations that both HCWs and CMs mentioned: a hesitancy to discuss attacks and normalization of violence. This normalization is a particularly concerning phenomenon which is prevalent in Colombia and in many other countries experiencing conflict such as Iraq [37]. Several interviewees in our study mentioned that because attacks were part of daily life, there was no real attempt to ameliorate them. This further reinforces the idea that after decades of conflict, people adapt to experiencing or witnessing violence but continue to suffer its legacy.

At the health system level, attacks frequently result in delays in healthcare access or, in some cases, a complete lack of medical care due to HCFs being temporarily closed, ambulances being unavailable, and missing equipment and medicines. Studies in Nigeria and South Sudan report similar experiences [33, 36].

Many HCWs reported being unhappy in their positions because of the constant threats to their safety. Others discussed their exhaustion, stress, anxiety, and the enormous cost to their families because of their profession. These tensions frequently result in attrition– several HCWs in this study reported that they moved or quit their jobs due to the working conditions. The personal impacts, compounded with the destabilization of facilities and services results in further strain on the healthcare system and negatively impacts patient care.

### Study insights must be operationalized

A final point that was crystallized in the analysis was the enormous importance of healthcare services in these communities and the critical need to make all stakeholders aware of the legal protections and clinical ramifications of attacks. While 83% of HCWs were aware of the Misión Médica and the protections required by law, only 38% of CMs reported familiarity with the Misión Médica. The Colombian government has made enormous efforts to achieve this level of awareness–it is likely that in most countries in conflict, many community members will have no awareness of international protections of healthcare. Nonetheless, ongoing Misión Médica campaigns to inform community members and especially non-state armed groups must be strengthened and expanded. Moreover, the findings of this study suggest that mere awareness of the law is inadequate for protection. Understanding the impacts of attacks, and how they undermine health services and access, and deplete health workers is a critical component of education. We hope that this paper supports this process and sheds light on the impacts of attacks in a way that motivates stakeholders to act.

### Strengths and limitations

Given the long history of conflict in Colombia and the complexity of attacks on health that are described in this paper, no one study can fully characterize all attacks, or their impact. The strengths include the inclusion of a diverse set of participants, including women health workers, technicians, and mid-level providers as well as community members in the sampling frame. By including categorical questions and open-ended questions in our interview protocol, the study allowed for both a descriptive analysis and qualitative one.

However, this study has some important limitations. Due to the small sample size in this survey project, the results may not be representative of the perspectives of all HCWs or CMs in Colombia. Since all CMs sampled were in some way related to the HCF study sites, there may have been an overestimation in the level of awareness of the MM. Also, because of the small sample size, the results of the quantitative analysis were not tested for statistical significance. In asking about experiences with attacks in the past, there may be respondent recall bias and because all participants did not directly witness or experience violence, there could be hearsay and confirmation bias in their responses. Since interviewers hand wrote notes from the interviews rather than audio recording, recording bias or error could have affected the accuracy of the participant quotes and responses. Moreover, because of safety concerns, we did not ask interviewees to identify the armed groups in any way, which, consequently, did not allow for disaggregated in depth perpetrator analysis. This limited our results in terms of understanding attack motivation. Due to the fear surrounding discussions of attacks on health in Colombia, some interviewees might have been hesitant to describe their full experiences. The study took place during the height of the COVID-19 pandemic in early 2021, and therefore, experiences with the virus and health services could have impacted interview responses, especially in over-sampling for COVID-19 related attacks. This study aimed to understand attacks by centering the health workers and community members that live through them but legal protections that build on these findings must consider the contexts and perpetrators involved. Finally, this was an exploratory study into the experiences of health workers and community members around violence against healthcare impacts and much work is needed to continue to understand these impacts and assess and address the vulnerabilities in health services in conflict-affected regions, both in Colombia and globally. Future studies could elaborate on comparing violence perpetrated by armed groups versus community members in conflict settings, develop more participant specific surveys for various groups, deepen qualitative analysis or expand the sample size of such studies to deepen our understanding of the impacts of violence against healthcare in Colombia.

## Conclusion

Beyond shedding light on the experiences of health workers and community members regarding attacks on health, this study aims to impact policy and action in Colombia. This study suggests that the current attacks on healthcare in Colombia need to be viewed as an extension of the five decade-long conflict and that, even as we move away from the official conflict, there is still a high incidence of assaults, particularly among the civilian population which needs to be addressed. Fear among both HCWs and CMs hinders attack reporting and negatively impacts both the general quality of life in the area and the quality of healthcare.

The study responses indicate clear directions for tangible efforts to prevent and protect against ongoing violence, mitigate the impacts, and hold perpetrators accountable. There needs to be an increased educational campaign for community members to humanize HCWs and foster understanding and respect for the healthcare system based on our study findings of community and armed group based violence. HCWs require psychosocial support to manage emotional distress, which could be provided in the form of professional therapy sessions, community groups, or even social media networks. HCWs need protection while working, especially those who are employed in traveling clinics where many participants felt an increased risk. This includes ensuring adequate staffing, improving management of security and risk, and increasing collaborations with public and private organizations. Finally, a more transparent and verifiable process needs to be put in place to make sure that people, especially HCWs, who have been targeted in an attack, are empowered to speak up so that the responsible parties may be held accountable.

These efforts must be made at both the local and national scales to improve the existing struggles of local health workers in Colombia. Accountability has been limited but the documentation of attacks in this study could contribute to drawing more attention to how catastrophic violence is for already fragile health systems and motivate stakeholders to prioritize protection.

In the future, more research is required into what intervention programs work best to mitigate community-perpetrated attacks. At the same time, it is also critical that more is known about specific motivations for attacks, perpetrator identity, and increased details to pinpoint the activity of armed groups that can lead to more directed advocacy work. Lessons and best practices from the Misión Médica program could be extended across borders to other countries experiencing similar challenges. The partnership between the Ministry of Health, civil society and medical groups was critical to the founding of the Misión Médica campaign and might be a strong beginning for other programs. The combination of health worker training, public facing awareness building (i.e. billboards, TV, commercials, radio), and national-level policy engagement could also prove useful in other settings. While much remains to be done, the Misión Médica appears to be a sturdy foundation for bolstering HCW security, improving community relations, and being a genuine asset in the effort to mitigate attacks against health in Colombia.

## Data Availability

Descriptive data is available at: https://github.com/rohinihaar/Colombiasurvey2023. The survey template is available at: https://github.com/rohinihaar/RIAH-. The qualitative datasets presented in this manuscript are not readily available because of the sensitive nature of the data. There are ethical requirements that stipulate that qualitative data cannot be shared.

## Abbreviations

AUC: Autodefensas Unidas de Colombia, Self Defense Forces of Colombia
CM: Community member
CRUE: Centros Reguladores de Urgencias y Emergencias y Desastres, Regulatory Center for Emergencies
ELN: Ejército de Liberación Nacional, National Liberation Army
FARC: Las Fuerzas Armadas Revolucionarias de Colombia, Revolutionary Armed Forces of Colombia-People’s Army
FARC-EP: Las Fuerzas Armadas Revolucionarias de Colombia- Ejército del Pueblo, Revolutionary Armed Forces of Colombia-People’s Army
HCF: Healthcare facility
HCW: Healthcare worker
IHL: International Humanitarian Law
MM: Misión Médica

## References

[1] International Committee of the Red Cross. Respecting and Protecting Health Care in Armed Conflicts and in Situations Not Covered by International Humanitarian Law. 2012.

[2] Colombia | International. Center for Transitional Justice [Internet]. [cited 2022 Dec 8]. Available from: https://www.ictj.org/location/colombia.

[3] Health Care in Danger. : Making the Case [Internet]. Geneva, Switzerland: International Committee of the Red Cross; 2011 [cited 2022 Dec 8]. 22 p. Available from: https://shop.icrc.org/health-care-in-danger-making-the-case-print-en.html.

[4] Safeguarding Health in Conflict Coalition. Safeguarding Health in Conflict [Internet]. [cited 2023 Jan 21]. Available from: https://www.safeguardinghealth.org/.

[5] World Health Organization. Surveillance System for Attacks on Health care (SSA) [Internet]. [cited 2023 Jan 21]. Available from: https://extranet.who.int/ssa/Index.aspx.

[6] Insecurity Insight [Internet]. [cited 2023 Jan 21]. Insecurity Insight ». Available from: https://insecurityinsight.org/.

[7] Insecurity Insight. Health Care in Conflict Program [Internet]. 2021 [cited 2021 May 28]. Available from: http://insecurityinsight.org/projects/healthcare.

[8] Safeguarding Health in C, Insecurity Insight. Ignoring Red Lines: Violence against health care in conflict 2022 [Internet]. Washington (DC): Safeguarding Health in Conflict Coalition; 2023 Jun [cited 2023 Jun 15] p. 116. Available from: https://insecurityinsight.org/wp-content/uploads/2023/05/SHCC-Report-Ignoring-Red-Lines.pdf.

[9] Researching the Impact of Attacks on Healthcare (RIAH). project - Humanitarian and Conflict Response Institute - The University of Manchester [Internet]. [cited 2020 Nov 30]. Available from: https://www.hcri.manchester.ac.uk/research/projects/riah/.

[10] NRC [Internet]. [cited 2022 Dec 8]. Colombia’s bloody history. Available from: https://www.nrc.no/perspectives/2015/nr-4/colombias-bloody-history/.

[11] Colombian Armed Conflict [Internet]. Justice for Colombia. [cited 2022 Dec 8]. Available from: https://justiceforcolombia.org/about-colombia/colombian-armed-conflict/.

[12] Nussio E. Learning from Shortcomings: The Demobilisation of Paramilitaries in Colombia. J Peacebuilding Dev [Internet]. 2011 [cited 2022 Dec 8];6(2):88–92. Available from: https://www.jstor.org/stable/48603404.

[13] Colombia’s Killer Networks. : The Military - Paramilitary Partnership and the United States [Internet]. [cited 2022 Dec 8]. Available from: https://www.hrw.org/reports/1996/killer1.htm.

[14] Turkewitz J, Colombia Seeks Justice for War Atrocities Via New Court. The New York Times [Internet]. 2021 Mar 6 [cited 2022 Dec 6]; Available from: https://www.nytimes.com/2021/03/06/world/americas/colombia-court-war-crimes.html.

[15] Turkewitz J, Glatsky G. Colombia truth commission report: Colombia Panel’s Report Is a Step Toward Mending a Civil War’s Scars. The New York Times [Internet]. 2022 Jun 28 [cited 2022 Dec 6]; Available from: https://www.nytimes.com/live/2022/06/28/world/colombia-truth-commission-report.

[16] Leonardo L. Colombian Violent Conflict: A Historical Perspective. Int J World Peace [Internet]. 2019 Dec;36(4):53–83. Available from: https://go.gale.com/ps/i.do?id=GALE%7CA607710138&sid=googleScholar&v=2.1&it=r&linkaccess=abs&issn=07423640&p=AONE&sw=w&userGroupName=anon%7Ecf7b12e8.

[17] Restrepo J, Spagat M. Civilian Casualties in the Colombian Conflict: A New Approach to Human Security. CEPR Davidson Inst [Internet]. 2004; Available from: https://personal.rhul.ac.uk/uhte/014/HS%20in%20Colombia%20Civil%20Conflict.pdf.

[18] Bernard V, Nikolova, Mariya, Cornet, Gaetane, Pothelet E, editors. International Review of the Red Cross, Humanitarian debate: Law, policy, action Violence against health care, Part I: The problem and the law. 2013;95(889):246. Available from: https://www.icrc.org/en/doc/resources/international-review/review-889-violence-against-health-care-1/review-889-all.pdf.

[19] Ministerio de Salud. y Protección Social [Internet]. [cited 2022 Dec 6]. Misión Médica. Available from: https://www.minsalud.gov.co/salud/PServicios/Paginas/mision-medica.aspx.

[20] de Caldas DT S de. Dirección Territorial de Salud de Caldas. 2023 [cited 2023 Sep 16]. Centro Regulador de, Urgencias CRUE. Available from: https://saluddecaldas.gov.co/publicaciones/3914/centro-regulador-de-urgencias-crue/.

[21] Health services deal with a pandemic and violence in Colombia. 2021 Mar 14 [cited 2022 Dec 8]; Available from: https://www.icrc.org/en/document/health-services-deal-pandemic-and-violence-colombia.

[22] February, UBHRC| II. ArcGIS StoryMaps. 2021 [cited 2022 Dec 6]. Violence Against Health Care: Attacks During a Pandemic. Available from: https://storymaps.arcgis.com/stories/fd6a804a17b74f0aaa3d00b76b9ab192.

[23] Insecurity Insight. Attacked and Threatened: Health Care at Risk [Internet]. [cited 2023 Jan 21]. Available from: https://map.insecurityinsight.org.

[24] Haar RJ, Read R, Fast L, Blanchet K, Rinaldi S, Taithe B et al. Violence against healthcare in conflict: a systematic review of the literature and agenda for future research. Confl Health [Internet]. 2021 May 7 [cited 2022 Dec 8];15(1):37. 10.1186/s13031-021-00372-7. 10.1186/s13031-021-00372-7 PMC8103060 33962623

[25] Haar RJ. RIAH Manchester Survey Template [Internet]. Github; 2023. Available from: https://github.com/rohinihaar/RIAH.

[26] Stata Statistical Software (2021). Release 17. College Station.

[27] Fereday J, Muir-Cochrane E. Demonstrating Rigor Using Thematic Analysis: A Hybrid Approach of Inductive and Deductive Coding and Theme Development. Int J Qual Methods [Internet]. 2006 Mar 1 [cited 2022 Dec 6];5(1):80–92. 10.1177/160940690600500107.

[28] Proudfoot K. Inductive/Deductive Hybrid Thematic Analysis in Mixed Methods Research. J Mix Methods Res [Internet]. 2022 Sep 20 [cited 2022 Dec 6]; 10.1177/15586898221126816.

[29] Dedoose Version 9. 0.17, web application for managing, analyzing and presenting qualitative and mixed method research data [Internet]. Los Angeles, CA: SocioCultural Research Consultants, LLC; 2021. Available from: www.dedoose.com.

[30] Olmos-Vega FM, Stalmeijer RE, Varpio L, Kahlke R. A practical guide to reflexivity in qualitative research: AMEE Guide No. 149. Med Teach. 2022;1–11. 10.1080/0142159X.2022.2057287 35389310

[31] 11 years of violence against health care in Syria. - Syrian Arab Republic | ReliefWeb [Internet]. [cited 2022 Dec 6]. Available from: https://reliefweb.int/report/syrian-arab-republic/11-years-violence-against-health-care-syria.

[32] Colombia: health care in danger. 2022 Mar 24 [cited 2022 Dec 6]; Available from: https://www.icrc.org/en/document/colombia-health-care-danger.

[33] Joint Health Staff Survey: Protection of Health Care South Sudan [Internet]. International Rescue Committee. ; 2022. Available from: https://www.rescue.org/sites/default/files/2022-10/Joint_SS_Health_Survey_October2022_Update.pdf.

[34] ICRC institutional Health Care in Danger. strategy 2020–2022, Protecting health care from violence and attacks in situations of armed conflict and other emergencies [Internet]. International Committee of the Red Cross; Available from: https://healthcareindanger.org/wp-content/uploads/2020/10/ICRC-HCiD-strategy-2020-2022.pdf?_hsmi=238083391&_hsenc=p2ANqtz-9AgJzU7WojMnR2ugXQvjKzpVOqzJOmazoVswAFO-3GLDmH77NjJHrcxqXte56UGPgzB5kCP126UJrhiQ6Uo9y0rqS5vLgcLYFpd0pXWxlsvSpXbQQ.

[35] International Rescue Committee. 11 Years of Violence Against Health Care in Syria [Internet]. International Rescue Committee; 2022 [cited 2022 Dec 6]. Available from: https://reliefweb.int/report/syrian-arab-republic/11-years-violence-against-health-care-syria.

[36] Joint Health Staff Survey: Protection of Health Care in Northeast Nigeria [Internet]. International Rescue Committee. ; 2022. Available from: https://www.rescue.org/sites/default/files/2022-11/Joint_BAY_Health_Survey_October2022_VFOct22_0.pdf.

[37] Burnham G, Malik S, Dhari Al-Shibli AS, Mahjoub AR, Baqer AQ, Baqer ZQ et al. Understanding the impact of conflict on health services in Iraq: information from 401 Iraqi refugee doctors in Jordan. Int J Health Plann Manage [Internet]. 2012 [cited 2023 Jan 5];27(1):e51–64. Available from: https://onlinelibrary.wiley.com/doi/abs/10.1002/hpm.109110.1002/hpm.1091. 10.1002/hpm.1091 21638312

